# In Vitro Photoprotective, Anti-Inflammatory, Moisturizing, and Antimelanogenic Effects of a Methanolic Extract of *Chrysophyllum lucentifolium* Cronquist

**DOI:** 10.3390/plants11010094

**Published:** 2021-12-28

**Authors:** Chaoran Song, Laura Rojas Lorz, Jongsung Lee, Jae Youl Cho

**Affiliations:** 1 Department of Integrative Biotechnology, Sungkyunkwan University, Suwon 16419, Korea; songchaoran115@163.com (C.S.); laurisrl@gmail.com (L.R.L.); 2 Biomedical Institute for Convergence at SKKU (BICS), Sungkyunkwan University, Suwon 16419, Korea; 3 Department of Biocosmetics, Sungkyunkwan University, Suwon 16419, Korea

**Keywords:** skin aging, skin whitening, ROS, NF-κB, AP-1

## Abstract

UVB exposure causes DNA mutation and ROS generation, which lead to skin photoaging, skin wrinkling, skin sagging, and uneven skin pigmentation. ROS activate the NF-κB and MAPK signaling pathways leading to production of inflammatory molecules such as COX-2, collagen-degrading proteins such as matrix metalloproteinases (MMPs), and moisture-deficiency-related proteins such as hyaluronidases (HYALs). UVB exposure also induces irregular skin pigmentation though melanin overproduction, related to CREB transcription factor activity and transcription of melanogenesis genes. Here, we demonstrate that *Chrysophyllum lucentifolium* methanol extract (Cl-ME) has antioxidant activity; it dose-dependently decreased the expression of COX-2, MMP-1, MMP-9, HYAL-1, and HYAL-4 by downregulating the NF-κB (IKKα/β, IκBα) and MAPK (ERK, JNK, and p38) pathways and increased the expression of *Col1a1*, which encodes a protein important for maintaining skin elasticity. Cl-ME also showed promising antimelanogenic activity by decreasing the expression of CREB, a transcription factor, which in turn inhibited the expression of genes encoding tyrosinase, MITF, TYRP1, and TYRP2. In summary, a methanol extract of *C. lucentifolium* exhibited antiphotoaging and antimelanogenic activity and could be useful in the cosmeceutical industry.

## 1. Introduction

Skin degradation is caused by both intrinsic and extrinsic aging. Intrinsic skin aging is mainly due to genetic changes, while extrinsic aging occurs through continuous exposure to environmental agents [1]. Solar exposure is the most important extrinsic aging factor, and photoaging is caused by UV radiation. UV radiation can be divided into three types by wavelength: UVA (315–400 nm), UVB (280–315 nm), and UVC (100–280 nm) [2,3]. UVB is generally considered the most skin-damaging of the three because it causes sunburn, inflammation, and erythema and deeply penetrates the skin, resulting in DNA mutation and generation of radical oxygen species (ROS) [4]. ROS are involved in initiation and conduction of a cascade of signaling events that lead to skin photoaging, skin aging, and wrinkle formation [5].

The cascade of events that occurs in skin after exposure to UVB radiation and consequent ROS generation includes activation of NF-κB, a transcription factor that interacts with IκB-kinase (IKK), triggering phosphorylation of IκB, translocation of NF-κB into the nucleus, and subsequent expression of genes involved in the UVB-induced inflammatory response [6]. Recent studies have shown that activation of the mitogen-activated protein kinase (MAPK) pathway, which includes the activity of extracellular signal-regulated kinase (ERK), c-Jun N-terminal kinase (JNK), and p38, is strongly correlated with the process of skin aging [7,8]. In this case, skin aging occurs as a result of increased expression of the inflammatory protein cyclooxygenase-2 (COX-2) and the expression of collagenases such as matrix metalloproteinases (MMPs) and hyaluronic acid-degrading enzymes such as hyaluronidases (HYALs) [7,9]. Therefore, antioxidative compounds that prevent ROS formation and the consequent activation of those pathways are in high demand in the cosmeceutical industry.

Another desirable trait of antiaging compounds is an ability to inhibit collagen degradation by MMPs [10] and increase collagen levels in the skin [11]. Collagen is a fundamental component of the extracellular matrix (ECM) and plays a critical role in maintaining skin elasticity and strength, which are gradually lost as the skin ages [12]. Another important response to UV irradiation is production of melanin as a protective mechanism against solar damage [13]. However, overproduction of melanin can manifest as melasma, age spotting, and uneven skin pigmentation, all signs of skin aging [14]. Melanin is produced through a process called melanogenesis, in which the enzyme L-tyrosine is converted into L-dihydroxyphenylalanine (L-DOPA) through the action of the enzyme tyrosinase and is later converted to melanin through a series of subsequent oxidations [15]. Furthermore, microphthalmia-associated transcription factor (MITF) and tyrosinase related proteins 1 (TRP-1) and 2 (TRP-2) are involved in the production of melanin [16]. According to current beauty standards, white skin is a desirable trait; therefore, compounds that decrease melanin production are in high demand. 

*Chrysophyllum lucentifolium* is a plant commonly found in South America and is traditionally used as an edible fruit and construction material [17]. Although no traditional uses have been reported for *C. lucentifolium*, other species from the same family have been shown to affect various skin conditions. A study in rats, for example, revealed that *Chrysophyllum cainito* leaves might be beneficial for wound healing [18]. Another species from the Sapotaceae family, *Pradosia mutisii* [19], has been reported to have antiaging, antiwrinkling, and antimelanogenic effects. *Argania spinosa,* another species in the family Sapotaceae, is used in cosmeceutical preparations to moisturize the skin and hair [20]. The potential of species in the family Sapotaceae in cosmeceutical and pharmaceutical preparations prompted us to investigate *C. lucentifolium* for antiphotoaging or antimelanogenic activity that might be valuable in the cosmeceutical industry. To investigate whether this plant can be also applied as a promising cosmeceuticals, metanolic extract was prepared. Since it has been reported that methanol is the optimal solvent to extract the highest content of active principles such as phenolic and steroidal components [21], we employed this solvent for cellular and molecular studies. Indeed, it was found that the methanol extract of this plant (Cl-ME) shows high capacity of anti-inflammatory and antioxidant activities. Therefore, we further explored its effect on UVB-induced photoaging and α-melanocyte stimulating hormone (α-MSH)-mediated melanogenic responses.

## 2. Results

### 2.1. HPLC Analysis of Cl-ME and Its Cytotoxicity toward HaCaT, HDF, HEK293T, and B16F10 Cells

**Figure 1:**
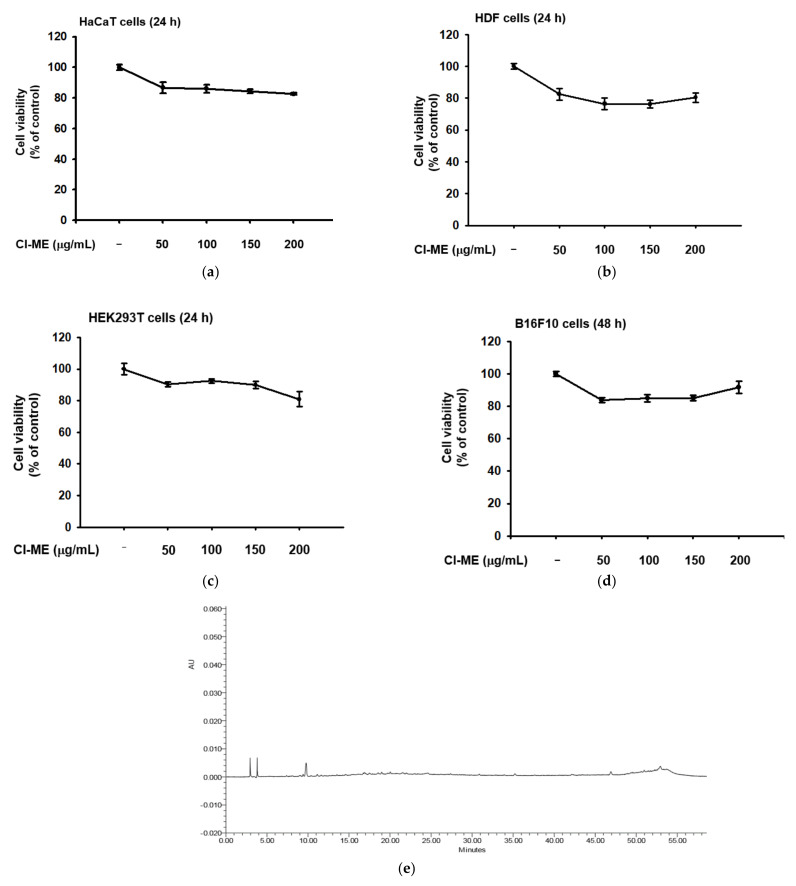
Compound analysis and cytotoxicity of Cl-ME toward HaCaT, HDF, HEK293, and B16F10 cells. (**a**) Viability of HaCaT, (**b**) HDF, (**c**) HEK293T, and (**d**) B16F10 cells after 24 h treatment with Cl-ME (0–200 µg/mL) was measured using the MTT assay. (**e**) The phytochemical fingerprinting of Cl-ME was observed by HPLC.

The cytotoxicity of a methanol extract of *C. lucentifolium* (Cl-ME) was tested in HaCaT, HDF, HEK293T, and B16F10 cells using the MTT assay. Cl-ME exhibited weak or slight cytotoxic activity at 150 and 200 µg/mL toward HaCaT (Figure 1a), HDF (Figure 1b), HEK293 (Figure 1c), and B16F10 (Figure 1d) cells less than 15 to 20%. To identify the active biochemical ingredients, we tested Cl-ME for luteolin, kaempferol, and quercetin, the well-known antioxidant compounds. However, according to HPLC analysis, none of these were present in Cl-ME (Figure 1e). 

### 2.2. Radical Scavenging Activity and Protective Effect of Cl-ME against Cell Damage from UVB and H_2_O_2_ in HaCaT Cells

**Figure 2:**
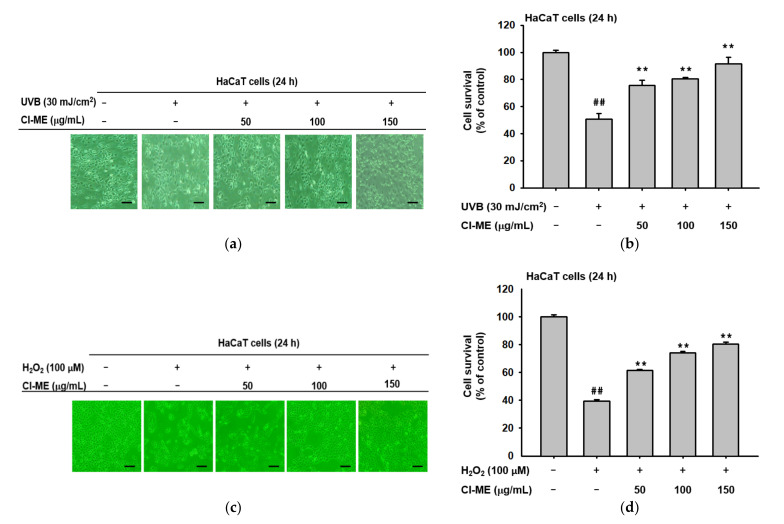

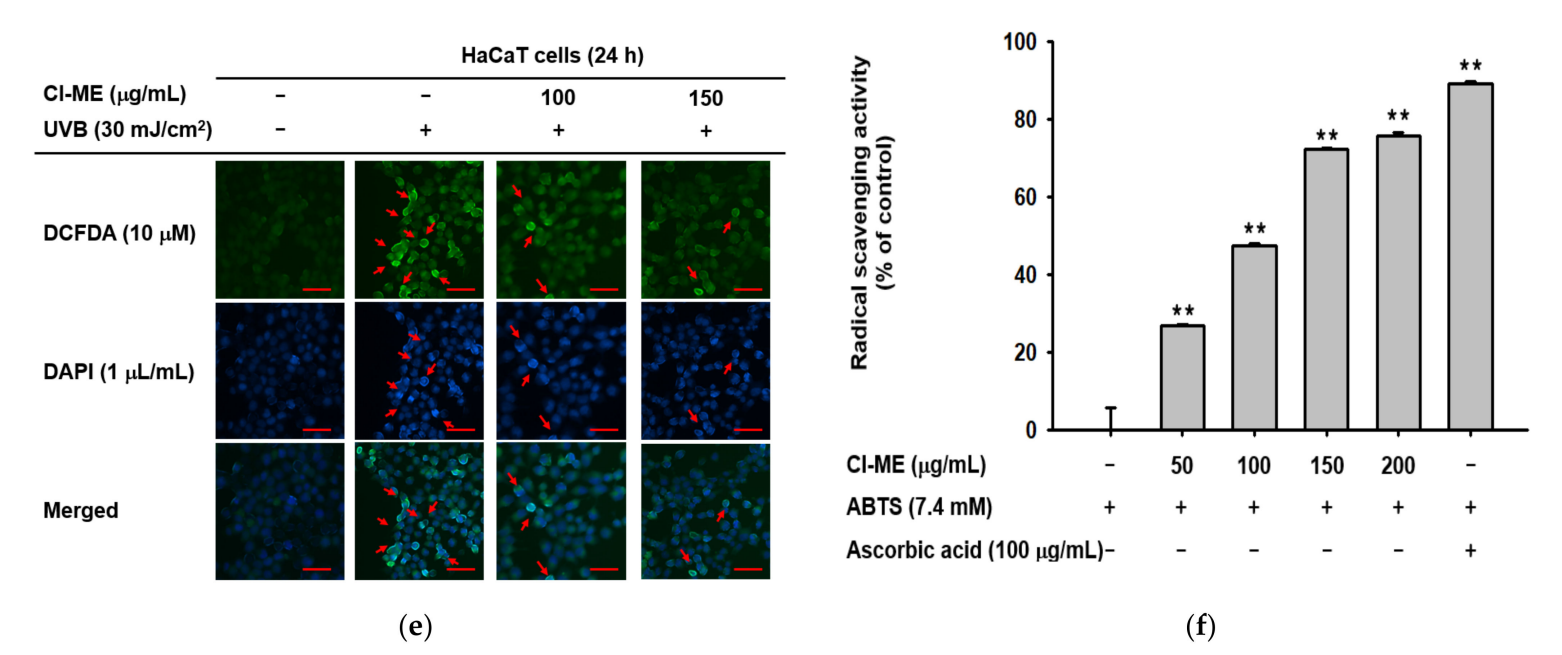
Antioxidant and photoprotective effects of Cl-ME on UVB- and H_2_O_2_-induced damage in HaCaT cells. (**a**,**c**) Morphological changes in UVB-treated (30 mJ/cm^2^) HaCaT cells after 24 h treatment with Cl-ME (0–150 µg/mL). (**b**,**d**) Survival (% of control) of UVB (**b**)- or H_2_O_2_ (**d**)-treated HaCaT cells after a 24 h treatment with Cl-ME. (**e**) Protective activity of Cl-ME against ROS and cell damage as measured through DCFDA (10 μM) and DAPI (1 μL/mL) staining in HaCaT cells after 24 h of treatment. (**f**) Radical scavenging activity of Cl-ME was measured using the ABTS assay with ascorbic acid as the positive control. ^##^ *p* < 0.01 compared to the normal group and ** *p* < 0.01 compared to the control group.

As previously explained, UVB exposure causes DNA mutation and ROS generation, which lead to photoaging, wrinkling, and sagging of skin [22,23]. Morphological analysis was employed to explore the protective effects of Cl-ME against UVB and H_2_O_2__._ As shown in Figure 2a,b, UVB irradiation induced morphological changes and approximately 60% cell death. Cl-ME treatment attenuated the UVB-induced cell damage in a dose-dependent manner and raised cell viability from 40% to over 80%. A similar pattern was observed upon H_2_O_2_ treatment (Figure 2c,d). Therefore, 150 µg/mL of Cl-ME was selected for subsequent experiments. Next, HaCaT cells were stained with H_2_-DCFDA and DAPI. UVB irradiation enhanced ROS generation and DNA damage, while Cl-ME alleviated ROS- and UVB-mediated cell damage (Figure 2e). We used the ABTS assay to explore the free radical scavenging ability of Cl-ME, with ascorbic acid as the positive control. Cl-ME exhibited dose-dependent radical scavenging activity (Figure 2f).

### 2.3. The Effect of Cl-ME on Moisture and Collagen 

**Figure 3:**
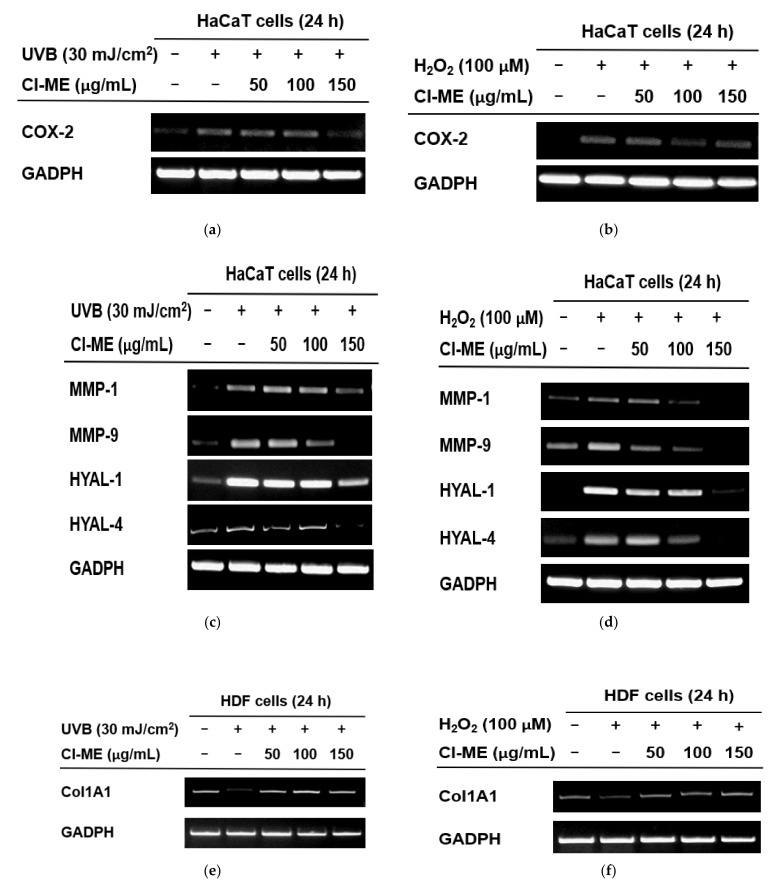

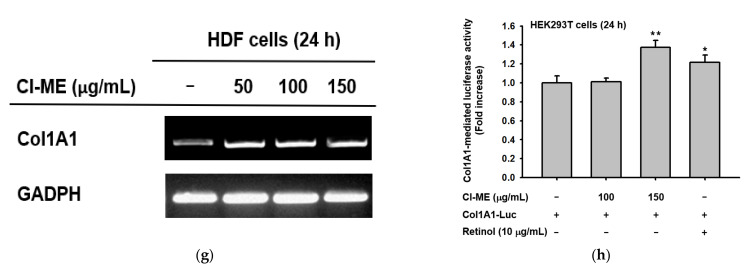
Prevention of moisture loss and collagen degradation and the effect of collagen formation of Cl-ME. (**a**,**b**) Expression of COX-2 in UVB (30 mJ/cm^2^)- or H_2_O_2_-(100 µM) treated HaCaT cells after 24 h treatment with Cl-ME. (**c**,**d**) Expression of MMP-1, MMP-9, HYAL-1, and HYAL-4 in HaCaT cells after a 24 h treatment with H_2_O_2_ and Cl-ME. (**e**,**f**) Recovery of Col1a1 expression in UVB-treated (30 mJ/cm^2^) or H_2_O_2_-treated HDF cells after 24 h of treatment with Cl-ME. (**f**) Recovery of Col1a1 expression in HDF cells after a 24 h treatment with H_2_O_2_ and Cl-ME. (**g**) Expression of the Col1a1 gene in HDF cells after a 24 h treatment with Cl-ME. (**h**) Col1a1-mediated luciferase activity in HEK293T cells, measured after 24 h of treatment with Cl-ME; retinol was used as the positive control. * *p* < 0.05 and ** *p* < 0.01 compared to the control group.

To examine the effect of Cl-ME on inflammatory markers, the expression of COX-2 was measured in the presence or absence of UVB (H_2_O_2_) or Cl-ME. Cl-ME dose-dependently reduced the expression of the COX-2 gene in both UVB (30 mJ/cm^2^) (Figure 3a)- and H_2_O_2_-treated (100 µM) HaCaT cells (Figure 3b). Matrix metalloproteinases and hyaluronidases degrade extracellular matrix proteins, leading to alterations such as skin wrinkling and aging [24]. UVB and H_2_O_2_ promoted MMP and HYAL activity, which accelerate the formation of skin wrinkles (Figure 3c,d). Meanwhile, Cl-ME dose-dependently reduced the production of MMP-1, MMP-9, HYAL-1, and HYAL-4 in UVB (30 mJ/cm^2^) (Figure 3c)- and H_2_O_2_-treated (100 µM) HaCaT cells (Figure 3d). Another molecule important in maintaining skin elasticity is collagen, which is the main component of the ECM and whose degradation diminishes skin flexibility, tensile strength, and ability to hold water [12]. In this case, Cl-ME not only attenuated the UVB (30 mJ/cm^2^)- and H_2_O_2_- induced decrease in Col1a1 expression in HDF cells (Figure 3e,f) but also showed an intrinsic ability to increase collagen levels in HDF cells (Figure 3g). Moreover, luciferase reporter assay showed that Cl-ME and retinol increased luciferase activity at the transcriptional level (Figure 3h). 

### 2.4. Cl-ME Downregulated the Inflammation-Associated NF-kB and AP-1 Pathways

**Figure 4:**
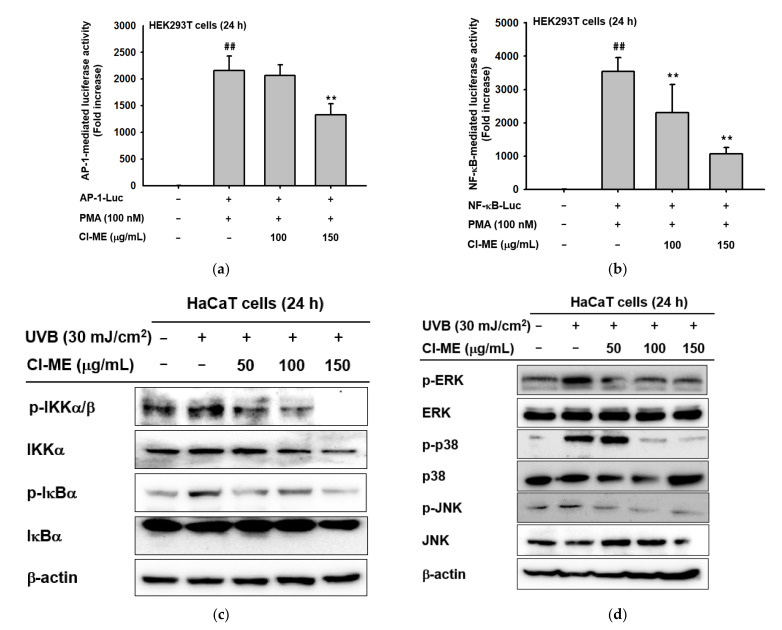
Cl-ME-mediated downregulation of the inflammatory NF-κB and AP-1 pathways. (**a**) NF-κB-mediated luciferase activity in PMA-activated (100 nM) HEK293T cells, measured after a 24 h treatment with Cl-ME (0–150 µg/mL). (**b**) AP-1-mediated luciferase activity in PMA-activated (100 nM) HEK293T cells, measured after a 12 h treatment with Cl-ME (0–150 µg/mL). (**c**) Phosphorylated and total forms of NF-κB pathway proteins (IKKα/β and IκBα) obtained from UVB-treated (30 mJ/cm^2^) HaCaT cells after a 24 h treatment with Cl-ME (0–150 µg/mL). (**d**) Phosphorylated and total forms of AP-1 pathway proteins (ERK, JNK, and p-38) from UVB-treated HaCaT cells after a 24 h treatment with Cl-ME (0–150 µg/mL). ^##^ *p* < 0.01 compared to the normal group and ** *p* < 0.01 compared to the control group.

UVB is responsible for activating numerous signaling cascades, such as the NF-κB and AP-1 pathways [25]. Initiation of the NF-κB and AP-1 is known to regulate the activity of MMPs and COX-2. We therefore investigated the effect of Cl-ME on NF-κB and AP-1 pathways. Cl-ME inhibited PMA-induced luciferase activity mediated by NF-κB and AP-1 (Figure 4a,b). Furthermore, to investigate the regulatory mechanism of Cl-ME, the phosphorylation level of proteins upstream of NF-κB (IKKα/β, IκBα) and AP-1(ERK, p38, JNK) was measured. Phosphor forms of IKKα/β, IκBα, ERK, p38, and JNK were dramatically induced by UVB irradiation and significantly suppressed by Cl-ME treatment in a dose-dependent manner (Figure 4d,e).

### 2.5. Antimelanogenic Effect of Cl-ME in B16F10 Cells through Downregulation of the CREB Pathway

**Figure 5:**
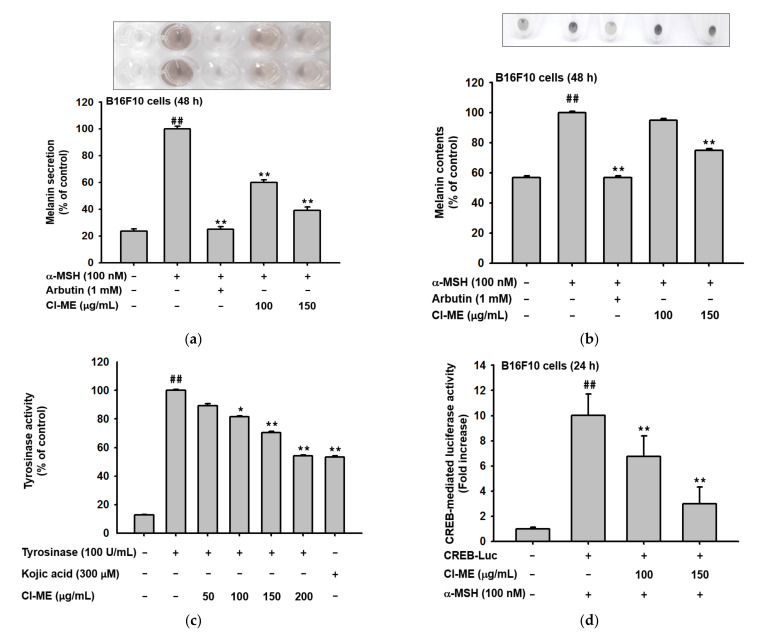

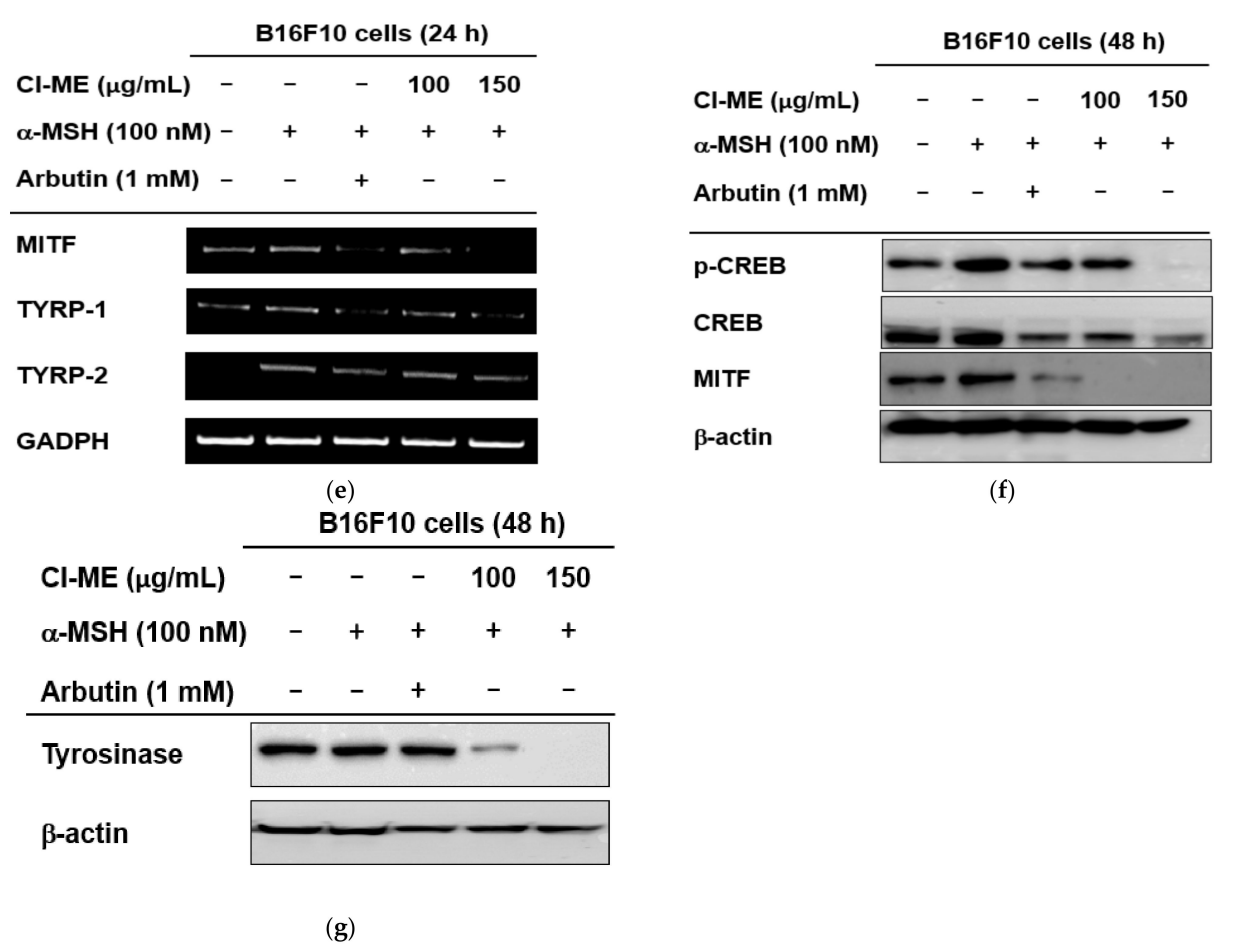
Antimelanogenic effect of Cl-ME in murine melanoma B16F10 cells. (**a**) Melanin secretion in B16F10 cells after 48 h of treatment with Cl-ME (0–150 µg/mL); arbutin (1 mM) was used as the positive control. (**b**) Melanin content in B16F10 cells after 48 h of treatment with Cl-ME (0–150 µg/mL) with arbutin (1 mM) as the positive control. (**c**) Tyrosinase activity assay of Cl-ME (0–200 µg/mL) with kojic acid (300 µM) as the positive control. (**d**) CREB-mediated luciferase activity in α-MSH-activated B16F10 cells after 48 h of treatment with Cl-ME (0–150 µg/mL). (**e**) Expression of MITF, TRP-1, and TRP-2 proteins in α-MSH-activated B16F10 cells after a 48 h treatment with Cl-ME (0–150 µg/mL). (**f**) Phosphorylated forms of CREB and total amounts of CREB and MITF in α-MSH-activated B16F10 cells after a 48 h treatment with Cl-ME (0–150 µg/mL); arbutin was the positive control. (**g**) Phosphorylated forms of tyrosinase obtained from α-MSH-activated B16F10 cells after a 48 h treatment with Cl-ME (0–150 µg/mL) using arbutin as the positive control. ^##^ *p* < 0.01 compared to the normal group, and * *p* < 0.05 and ** *p* < 0.01 compared to the control group.

Human skin pigmentation is controlled by a process called melanogenesis, which plays an important role in preventing skin injury from UV radiation [13]. To examine the effect of Cl-ME on melanogenesis, B16F10 cells were induced by α-MSH and then treated with Cl-ME or arbutin. Arbutin was used as a positive control because it inhibits melanin biosynthesis by decreasing tyrosinase activity. The level of melanin secreted was increased four-fold by α-MSH treatment but was reduced to about one-quarter by Cl-ME (150 µg/mL). The inhibitory effect of Cl-ME on α-MSH-stimulated melanin production was less dramatic. Cl-ME (150 µg/mL) treatment decreased the melanin content by 20%. To investigate whether Cl-ME-mediated antimelanogenesis is related to enzyme activity, the activity of tyrosinase, a vital kinase in melanin synthesis, was determined [26]. A mushroom tyrosinase assay illustrated that Cl-ME (150 µg/mL) treatment decreased tyrosinase activity by 30%; in comparison, kojic acid, a known tyrosinase inhibitor, suppressed tyrosinase activity by 43% [27]. Many signaling molecules modulate the melanogenic process, including tyrosinase enzyme, TYRPs, and their transcription factors, such as MITF and CREB [28]. Luciferase reporter assay showed that Cl-ME (150 µg/mL) decreased the transcriptional activity of CREB in a dose-dependent matter (Figure 5d). Furthermore, we observed a reduction in the expression of melanin production-related proteins such as MITF, TRP-1, and TRP-2 upon Cl-ME treatment (Figure 5e), as confirmed by western blots of CREB and MITF transcription factors (Figure 5f) as well as tyrosinase (Figure 5g).

## 3. Discussion

Plants in the genus *Chrysophyllum* are edible and have multiple health benefits. *Chrysophyllum abidum* fruit exerts anti-inflammatory activity by inhibiting NF-κB activation [29]. *Chrysophyllum caimito* stem bark extract was shown to have an antitumorigenic effect in hepatocellular carcinoma by promoting apoptosis of Hep G2 cells [30]. This study aimed to investigate if *Chrysophyllum lucentifolium,* a shrub in the genus *Chrysophyllum,* had a protective effect against UVB- or H_2_O_2_-induced cell damage and to explore the underlying mechanisms.

Our skin is exposed to ultraviolet light during outdoor activities, which can cause damage. Continuous exposure to UV can induce inflammation, resulting in production of a range of inflammatory cytokines and keratinocyte death. Excessive UV exposure triggers the accumulation of ROS, leading to oxidative stress, DNA mutation, skin aging, and development of skin cancers. A vital stage in choosing a compound suitable for use as a cosmeceutical compound is determining its cytotoxicity; minimal toxicity is critical for pharmaceutical and cosmetic formulations [31]. Cl-ME was toxic at concentrations of 200 µg/mL toward HaCaT (Figure 1a) and B16F10 cells (Figure 1d) but showed no significant cytotoxicity at the same concentration toward HDF (Figure 1b) and HEK293T cells (Figure 1c). Therefore, a concentration of 150 µg/mL of Cl-ME was chosen for all subsequent experiments. 

Antioxidant compounds inhibit ROS generation and impact skin aging positively. An important step in the search for promising antiaging compounds is to determine the presence or absence of antioxidant activity [32]. We tested Cl-ME for the presence of luteolin, kaempferol, and quercetin, which are well-known antioxidant compounds. HPLC analysis failed to detect the presence of these compounds in Cl-ME (Figure 1e). Therefore, its antioxidant, antimelanogenic, and antiaging activities are apparently due to other intrinsic compounds. Coumaric acid [19], spinasterol, 6-hydroxyflavanone, 4-dihydroxybenzoic acid, taraxerol, taraxerone, and lupeol acetate [32] have been found in plants in the Sapotaceae family and have antioxidant and antiaging effects. A more extensive analysis of compounds present in Cl-ME is required to identify components responsible for the antioxidant, antimelanogenic, and antiaging activity of this extract. 

As previously described, UVB exposure causes DNA mutation and ROS generation, which lead to photoaging, wrinkling, and sagging of skin [22,33]. Thus, a photoprotective compound should have inherent radical scavenging activity so that it can prevent cell damage and death caused by both UVB radiation itself and the UVB-induced generation of ROS. Consistent with previous studies, UVB exposure and H_2_O_2_ treatment resulted in the death of a large number of keratinocytes as well as morphological changes. Cl-ME had a photoprotective effect; it decreased the percentage of dead cells (Figure 1b,d). Additionally, Cl-ME had significant, dose-dependent ROS and cell-damage inhibitory activity in UVB-treated HaCaT cells (Figure 2e). Moreover, Cl-ME had important, dose-dependent ABTS radical scavenging activity (Figure 2f). Thus, it helped to prevent both ROS- and UVB-mediated damage to cells.

UV radiation increases the expression of COX-2, MMP, and HYAL in the skin, all of which play an important role in skin photoaging. UVB-mediated overexpression of COX-2, for example, causes inflammation, itching, and oxidative DNA damage, which eventually lead to photoaging symptoms [22,34]. An increase in the expression of MMPs causes skin photoaging through degradation of ECM proteins such as collagen, elastin, and fibronectin [24]. UVB-mediated overproduction of hyaluronidases plays a part in skin aging as well because degradation of hyaluronic acid, a water-retaining molecule important in maintaining skin hydration, leads to skin wrinkling, sagging, and generalized aging [35]. Cl-ME reduced the expression of COX-2 in both UVB- and H_2_O_2_-treated (100 µM) HaCaT cells (Figure 3a,b). The antiaging effect of Cl-ME was not only observed through its ability to decrease the levels of MMPs and HYALs upon UVB and H_2_O_2_ stimulation, but also its ability to promote collagen synthesis (Figure 3e–g). All these results suggest that Cl-ME is potentially useful in the cosmeceutical industry as an anti-inflammatory and antiaging compound.

AP-1, a transcription factor, is activated by upstream molecules (ERK, JNK, p38), while NF-κB is activated by IKKα/β and IκBα. Upon UVB irradiation, AP-1 and NF-κB are activated and induce the production of a series of cascade mediators. For instance, MMPs and COX-2 degrade collagen and other ECM components in the skin [9] and cause inflammatory responses that contribute to skin aging [36]. Because activation of those pathways is closely related to skin aging, Cl-ME is a promising antiaging compound because it dose-dependently attenuated the expression of signaling molecules in the AP-1 and NF-κB pathways based on luciferase assays and immunoblotting (Figure 4). Our results demonstrated that the anti-inflammatory and antiaging effects of Cl-ME are mediated by its targeting of the AP-1 and NF-κB pathways.

Overactivated melanogenesis can damage DNA and other molecules, alter immunological responses, and cause uneven skin pigmentation and aging [28]. Melanin production is closely related to activation of CREB transcription by α-MSH, which activates MITF and results in the expression of melanin-producing proteins such as TRP-1, TRP-2, and tyrosinase [28]. Current cosmetic trends have increased the demand for compounds that can attenuate melanogenesis. Cl-ME is a candidate for whitening cosmeceutical preparations because it decreased both melanin secretion and melanin content in B16F10 cells, diminished the activity of tyrosinase, and decreased the transcriptional activation of CREB. Additionally, expression of MITF, TRP-1, and TRP-2 genes and p-CREB, MITF, and tyrosinase proteins was blocked by Cl-ME (Figure 5). All these results confirm that Cl-ME has significant antimelanogenic activity and thus high potential value in the cosmeceutical industry.

Unfortunately, there was no report on phytochemical study with *Chrysophyllum lucentifolium*. Surprisingly, HPLC analysis with standard flavonoids such as luteolin, kaempferol, and quercetin did not show any significantly identified levels of these compounds (Figure 1e), implying that major principles are not related to simple flavonoid backbones, and further phytochemical studies are needed. However, chemical analysis with *Chrysophyllum* plants such as *C. lacourtianum, C. oliviforme,* *C. cainito*, *C. albidum,* and *C. pruniforme* have been reported [37,38,39,40]. A variety of compounds, including 3-galloyl myrecetrin, rutin, quercetrin, myrecetrin, myricetin, quercetin, isoquercitrin, kaempferol, caffeic acid, trans-ferulic acid, gallic acid, stigmasterol, epicatechin, epigallocatechin, procyanidin B5, (+)-catechin, (-)-epicatechin, (+)-gallocatechin, (-)-epigallocatechin, β-amyrin, lupeol, 2,5-dimethylpyrazine, 3,5-dihydroxy-6-methyl-2,3-dihydropyran-4-one, indole, norharman, and methyl petroselinate were identified from these plants [37,38,39,40]. Of them, interestingly, catechin and its analogs are known to be active principles showing antimelanogenic, anti-inflammatory, and antiwrinkle formation activities during UVB irradiation [41,42,43], although quercetin and its analogs are also known to be other major components with antiphotoprotective effects by radical scavenging activity [44,45]. Based on HPLC profile and previous reports, it is expected that catechin and its related compounds could be included in this plant. To prove this, we will further examine these compounds by LC-MS/MS analysis and activity-guided fractionation.

## 4. Materials and Methods

### 4.1. Materials

*C. lucentifolium* methanolic extract (Cl-ME) was obtained from the Korean Plant Extract Bank (Daejeon, Korea, http://extract.kribb.re.kr, accessed on 1 October 2021). Streptomycin, penicillin, phorbol-12-myristate-13 acetate (PMA), fetal bovine serum (FBS), Dulbecco’s Modified Eagle’s Medium (DMEM), and phosphate-buffered saline (PBS) were purchased from Gibco (Grand Island, NY, USA). Hydrogen peroxide (H_2_O_2_), polyethyleneimine (PEI), 2,2′-azino-bis (3-ethylbenzothiazoline-6-sulphonic acid) diammonium salt (ABTS), kojic acid, mushroom tyrosinase, arbutin, α-melanocyte stimulating hormone (α-MSH), L-DOPA ethyl ester, ascorbic acid, DAPI stain, DCFDA stain, and other chemical ingredients were purchased from Sigma Aldrich. Specific primers for semiquantitative RT-PCR were synthetized by Macrogen (Seoul, Korea), and a cDNA synthesis kit was purchased from Bio-D Inc. (Seoul Korea). Phosphorylated and total forms of IKK α/β, IκBα, ERK, p38, and JNK were obtained from Cell Signaling Technology (Beverly, MA, USA), and phosphorylated forms of MITF, tyrosinase, β-actin, and CREB and the total form of CREB were purchased from Santa Cruz Biotechnology (Dallas, TX, USA). Cell lines (HEK293.T, B16F10, HaCaT, and HDF cells) used in this study were obtained from American Type Culture Collection (Rockville, MD, USA).

### 4.2. High-Performance Liquid Chromatography (HPLC)

To determine the compound present in Cl-ME, an Agilent 1260 HPLC system was employed. Luteolin, quercetin, and kaempferol were used as standard compounds in high-performance liquid chromatography. Separation was performed using a C18 reverse-phase chromatography column (250 mm × 4.6 mm, 5 µm). Mobile phase A was 0.1% acetic acid, and mobile phase B was 100% acetonitrile. Constant flow rate was 1.0 mL/min with gradient elution. Injection volume was 10 µL. UV absorbance was monitored at 350 nm, as described previously [46].

### 4.3. Cell Culture

HaCaT cells and B16F10, HDF, and HEK293T cells were cultured in media with 10% or 5% FBS, respectively. All cells were maintained in DMEM with 1% penicillin–streptomycin at 37 °C in a 5% humidified incubator.

### 4.4. Cell Viability Assessment

HaCaT, HDF, B16F10, and HEK293T cells were seeded in 96-well plates. After a 24 h incubation, cells were treated with Cl-ME (0–150 µg/mL) for 24 h or 48 h. The viability of each cell line was assessed using the MTT assay.

### 4.5. ABTS Radical Scavenging Activity

To determine the radical scavenging activity of Cl-ME, the ABTS assay was performed as reported previously [47]. A mixture of 7.4 mM ABTS and 2.4 mM potassium persulfate was prepared and incubated at room temperature overnight. Indicated doses of Cl-ME (0–150 µg/mL) or ascorbic acid were reacted with ABTS solution. The mixture was transferred to a 96-well plate and incubated at 37 °C for 30 min. The absorbance at 730 nm was read, and the ABTS scavenging effect was calculated as follows:

ABTS scavenging effect (%) = (A_1_−A_0_)/A_0_ × 100

where A_0_ is the absorbance of ABTS, and A_1_ is the absorbance [48].

### 4.6. Post UVB and H_2_O_2_ Treatment Morphological Evaluation

For UVB and H_2_O_2_ treatment, HaCaT cells were seeded in a 6-well plate at a density of 2 × 10^5^ cells/mL, incubated with Cl-ME for 30 min, washed with PBS, and exposed to either H_2_O_2_ (100 µM) or UVB radiation from a UVB lamp (30 mJ/cm^2^). After H_2_O_2_ or UVB treatment, cells were treated with Cl-ME (0–150 µg/mL) for 24 h. As previously reported [49], morphological changes and cell quantity were assessed using a microscope and ImageJ software. Cell survival was determined as follows:

Cell survival (% of control): A_1_/A_0_ × 100

where A_1_ refers to treated cells and A_0_ to normal untreated cells.

### 4.7. Semi-Quantitative RT-PCR Analysis

Cells were treated with UVB and H_2_O_2_ as described above. Cl-ME (0–150 µg/mL) was then added to cells for 24 h. To measure the expression of melanin production-related genes, B16F10 cells were seeded at a density of 1 × 10^5^ cells/mL in 12-well plates. After 24 h, cells were treated with α-MSH (100 nM), Cl-ME (0–150 μg/mL), or arbutin (1 mM), followed by a 24 h incubation. Total mRNA was isolated using TRIzol reagent according to the manufacturer’s instructions [48]. cDNA was synthesized using a cDNA synthesis kit, and reverse transcription-polymerase chain reaction (RT-PCR) was conducted using the primers listed in Table 1 and Table 2.

### 4.8. ROS and DAPI Staining

HaCaT cells were treated as explained above. After UVB treatment, cells were washed with PBS, stained with 10 µM DCFDA, and incubated for 30 min in the dark. Then, cells were fixed in formaldehyde solution (100 µL/mL) and prepared for DAPI staining. ROS-stained and fixed cells were washed twice with PBS, stained with DAPI (1 µL/mL), and maintained in the dark for 20 min, as described previously [19].

### 4.9. Melanin Secretion and Content Assay

B16F10 cells were treated with α-MSH (100 nM), Cl-ME (0–150 μg/mL), or arbutin (1 mM) and incubated for an additional 48 h. Determination of melanin secretion and content was conducted according to a previous report [50]. Pictures were taken to assess melanin production as a color change, and melanin secretion and melanin content were measured at an absorbance of 475 nm and 405 nm, respectively. Both melanin secretion and content were expressed as percentage, as follows:

Melanin secretion/melanin content % = A_1_/A_0_ × 100

where A_1_ is the mean absorbance of the sample, and A_0_ is the mean absorbance of the control group.

### 4.10. Tyrosinase Assay

For the tyrosinase assay, 50 μL of 6 mM L-DOPA (previously dissolved in 50 mM potassium phosphate buffer, pH 6.8) was combined with 50 μL of Cl-ME or kojic acid and reacted for 15 min at room temperature. Afterward, 50 μL of mushroom tyrosinase (100 units/mL) was added, and the absorbance of the mixture was measured at 475 nm. Tyrosinase activity was calculated as follows:

Tyrosinase activity % = A_1_/A_0_ × 100

where A_1_ is the mean absorbance of the sample, and A_0_ is the mean absorbance of the control group.

### 4.11. Plasmid Transfection and Luciferase Reporter Gene Assay

HEK293T and B16F10 cells were seeded in 24-well plates. Transfection with luciferase-expressing genes (*NF-κB*, *AP-1*, and *CREB*) was carried out using the PEI method for 24 h. Cells were incubated with different concentrations of Cl-ME for 24 h. Absorbance was measured using a luciferase assay system, and a β-galactosidase construct (0.1 µg/well) was used as the control.

### 4.12. Western Blot Analysis

HaCaT and B16F10 cells were treated with Cl-ME (0–150 µg/mL) for 30 min, washed with PBS, and subjected to UVB treatment (30 mJ/cm^2^). After UVB treatment, cells were washed with PBS and treated for 24 h with Cl-ME (0–150 µg/mL). For B16F10 cells, induction was carried out as explained previously. Cell lysates were prepared and then subjected to sodium dodecyl sulfate (SDS)-polyacrylamide gel electrophoresis and transferred to polyvinylidene fluoride membranes. Using specific antibodies, total and phosphorylated forms of target proteins were detected and visualized using chemiluminescent reagents [51]. 

### 4.13. Statistical Analysis

All data are presented as mean ± standard deviation (SD) of at least three independent experiments. A Mann–Whitney test was used to compare statistical differences between the experimental and control groups, and a *p* value < 0.05 was considered statistically significant (* *p* < 0.05, ** *p* < 0.01). All statistical analyses were conducted using SPSS (SPSS Inc., Chicago, IL, USA).

## 5. Conclusions

**Figure 6:**
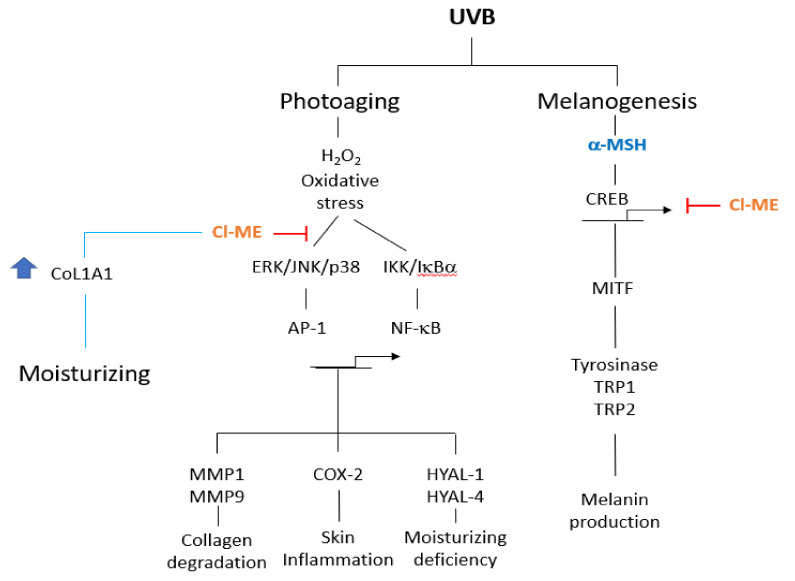
Schematic pathway illustrating the moisturizing, antiaging, anti-inflammatory, and antimelanogenic effects of Cl-ME in vitro. Upon UVB, H_2_O_2_, or α-MSH stimulation conditions, AP-1, NF-κB, and CREB pathways are initiated. By these pathways, expression of MMPs, COX-2, HYALs, and tyrosinase is increased, leading to skin inflammation, collagen degradation, and moisturizing deficiency, as well as pigmentation. Cl-ME plays its role in moisturizing, antiaging, anti-inflammatory, and antimelanogenic effects by targeting AP-1, NF-κB, and CREB pathways.

In summary, we demonstrated that Cl-ME has significant antioxidant activity, antiaging effects, and anti-inflammatory effects due to dose-dependent inhibition of the expression of COX-2, MMP-1, MMP-9, HYAL-1, and HYAL-4 through downregulation of NF-κB (IKKα/β, IκBα) and AP-1 signaling pathway proteins (ERK, JNK, p38), as summarized in Figure 6. In addition, Cl-ME increased the expression of Col1a1, which is important for maintaining skin elasticity. Furthermore, Cl-ME showed promising antimelanogenic activity by decreasing the expression of CREB, which resulted in a reduction in the expression of melanogenesis-related genes such as tyrosinase, MITF, TRP-1, and TRP-2. We conclude that Cl-ME is a potentially valuable cosmeceutical ingredient due to its antiaging and antimelanogenic activities.

## Figures and Tables

**Table 1:** Sequences of human primers used in semiquantitative RT-PCR.

Name	Primer	Sequence (5′ to 3′)
MMP-1	Forward	TCTGACGTTGATCCCAGAGAGCAG
	Reverse	CAGGGTGACACCAGTGACTGCAC
MMP-9	Forward	GCCACTTGTCGGCGATAAGG
	Reverse	CACTGTCCACCCCTCAGAGC
HYAL-1	Forward	CAGAATGCCAGCCTGATTGC
	Reverse	CCGGTGTAGTTGGGGCTTAG
HYAL-4	Forward	TGAGCTCTCTTGGCTCTGGA
	Reverse	AGGCAGCACTTTCTCCTATGG
COX-2	Forward	GGGATTTTGGAACGTTGTGAA
	Reverse	CGACATTGTAAGTTGGTGGACTGT
Col1A1	Forward	CAGGTACCATGACCGAGACG
	Reverse	AGCACCATCATTTCCACGAG
GADPH	Forward	GCACCGTCAAGGCTGAGAAC
	Reverse	ATGGTGGTGAAGACGCCAGT

**Table 2:** Sequences of mouse primers used in the semi-quantitative RT-PCR.

Name	Primer	Sequence (5′ to 3′)
MITF	Forward	AACTCSTGCGTGAGCAGATG
	Reverse	TACCTGGTGCCTCTGAGCTT
TRP-1	Forward	ATGGAACGGGAGGACAAACC
	Reverse	TCCTGACCTGGCCATTGAAC
TRP-2	Forward	CAGTTTCCCCGAGTCTGCAT
	Reverse	GTCTAAGGCGCCCAAGAACT
GADPH	Forward	ACCACAGTCCATGCCATCAC
	Reverse	CCACCACCCTGTTGCTGTAG

## Data Availability

The data is contained within the article.

## Abbreviations

Cl-ME	*Chrysophyllum lucentifolium* methanol extract
COX-2	Cyclooxygenease-2
CREB	cAMP Response element (CRE)
DAPI	4′,6-Diamidino-2-phenylindole
DCDFA	2′,7′-Dichlorodihydrofluorescein diacetate
ERK	Extracellular signal-regulated kinase
JNK	c-Jun N-terminal kinase
MAPKs	Mitogen-activated protein kinases
MMPs	Matrix metalloproteinases
MITF	Microphthalmia-associated transcription factor
α-MSH	α-Melanocyte-stimulating hormone
MTT	3-(4,5-Dimethylthiazol-2-yl)-2,5-diphenyltetrazolium bromide
ROS	Reactive oxygen species
RT-PCR	Reverse transcription-polymerase chain reaction
TYRP-1	Tyrosinase related protein 1
TYRP-2	Tyrosinase related protein 2
UV	Ultraviolet
